# A Single-Centre Experience of the Management and Surgical Outcomes of Late-Onset Idiopathic Aqueductal Stenosis

**DOI:** 10.7759/cureus.60168

**Published:** 2024-05-12

**Authors:** Belal Mohamed, Mohamed Okasha, Ian Coulter, Damian Holliman

**Affiliations:** 1 Neurosurgery, Ninewells Hospital, Dundee, GBR; 2 Neurosurgery, Royal Victoria Infirmary, Newcastle Upon Tyne, GBR

**Keywords:** late onset, csf, hydrocephalus, endoscopic third ventriculostomy, aqueductal stenosis

## Abstract

Background

Although idiopathic aqueductal stenosis is a congenital disorder, some patients present in adulthood. Many theories have tried to account for the late-onset presentation; however, the aetiology remains uncertain. This study aimed to investigate the clinical presentation, management, and outcomes of patients with late-onset idiopathic aqueductal stenosis (LIAS) managed at our centre.

Methodology

A retrospective study of patients with a diagnosis of LIAS managed at our centre between 1996 and 2018 was undertaken. Data on clinical presentation, imaging, management, and outcomes were retrieved from patient records and radiology reports.

Results

A total of 20 patients were diagnosed with LIAS during the study period. Endoscopic third ventriculostomy (ETV) was the initial modality of treatment for nine patients, ventriculoperitoneal shunt (VPS) for four patients, and conservative management in seven patients, in four of them intracranial pressure (ICP) was found to be normal following a period of ICP monitoring. The median follow-up period was three years (1 month to 24 years). One patient was lost to follow-up. One ETV failed in the first six months necessitating VPS insertion. Two cases that were initially managed conservatively required a VPS three and nine years following the initial presentation. Of the patients undergoing VPS insertion, all subsequently required valve adjustment or surgical revision.

Conclusions

The majority of patients with LIAS undergoing ETV were managed successfully, whereas VPS insertion was associated with a high rate of revision surgery in this cohort. ETV should be considered as the treatment of choice to avoid the long-term complications of shunting for patients with LIAS.

## Introduction

Aqueductal stenosis (AS) has many different aetiologies, including neoplasia and post-haemorrhagic and post-infectious obstruction [1,2]. AS is responsible for 6%-66% of cases of hydrocephalus in children and 5%-49% in adults [3]. In approximately 75% of patients, the aetiology is not known (idiopathic aqueductal stenosis (IAS)) [4,5]. Adult-onset AS without an apparent obstructive cause is termed late-onset idiopathic aqueductal stenosis (LIAS), and its incidence is not known [6,7].

While the underlying aetiopathogenesis remains uncertain, various theories have been proposed. Venous hypertension has been postulated as a mechanism responsible for ventricular dilatation and subsequent aqueductal obstruction [7]. Increasing resistance to cerebrospinal fluid (CSF) outflow through the extracellular space of the brain due to deep white matter ischaemia in late adulthood has also been purported to contribute to the development of LIAS [8].

Patients with LIAS present with various clinical symptoms, including headache, visual disturbance, dementia, gait disturbances, seizures, and, rarely, spontaneous CSF rhinorrhea [2,9]. In suspected cases, magnetic resonance imaging (MRI) is the radiological modality of choice to (a) demonstrate tri-ventricular dilation and (b) observe CSF flow obstruction at the level of the aqueduct. In particular, the fast imaging employing steady-state acquisition (FIESTA)/constructive interference in steady-state (CISS) sequences illustrates the CSF-parenchymal interface well to allow the discovery of obstructive anatomy such as arachnoid webs. Other features observed on MR scanning typically include downward or anterior bulging of the floor of the third ventricle and the absence of a space-occupying lesion [10]. Significantly symptomatic cases necessitate CSF diversion via an endoscopic third ventriculostomy (ETV) or CSF shunting, typically a ventriculoperitoneal shunt (VPS).

We aimed to perform a retrospective analysis of patients with LIAS managed at our unit to further understand the presenting features, the efficacy of treatment strategies, and the long-term outcomes for patients with this uncommon condition.

## Materials and methods

The study was registered and approved as a service evaluation study at our institution. Patients aged 18 years and older (defined in the United Kingdom as adults) referred to or admitted to the neurosurgery department of the Royal Victoria Infirmary or Newcastle General Hospital between 1996 and 2018 were included. These patients with a new diagnosis of symptomatic LIAS and no previous manifestations suggesting early-onset AS were included in the analysis. Eligible patients were identified via electronic records, departmental databases, and hospital records. Data on clinical presentation, brain imaging, treatment, and outcomes (assessed during the follow-up period) were obtained from patient records, electronic databases, and formal radiological reports. Chronic symptoms were defined based on symptom duration ≥6 months, in keeping with the previous work by Fukuhara and Luciano [9].

Patients with a history dating back to childhood, or who received treatment for AS before the age of 18 years and those with adult-onset AS secondary to an identifiable cause, such as an obstructive lesion or intracranial haemorrhage, were excluded from the analysis. A diagnosis was made by the patient’s responsible clinician based on the pertinent radiological findings of tri-ventriculomegaly, AS, and the absence of compressive pathology.

Computerised tomography (CT) scanning was the predominant diagnostic tool utilised in our department to investigate patients with suspected hydrocephalus until 2008. Thereafter, MRI was more readily available and commonly employed to assess ventricular anatomy, exclude potentially compressive lesions at the level of the aqueduct, and, in some cases, for the dynamic assessment of CSF flow through the aqueduct (using phase-contrast CINE sequences). The Evans ratio was calculated where possible utilising imaging undertaken at the time of diagnosis (the ratio of the maximum width of the frontal horns of the lateral ventricles to the maximal internal biparietal diameter of the skull at the same level).

Treatment was at the discretion of the responsible surgeon and based on clinical assessment, degree of ventriculomegaly, radiologic evidence of intracranial pressure (ICP), and, in equivocal cases, ICP monitoring. Surgical treatment included the insertion of a VPS or ETV. In equivocal cases with mild symptoms or normal ICP monitoring, conservative treatment in the form of symptomatic management along with clinical and imaging surveillance was offered. Patients who underwent ETV were followed up by routine head MRI scans one to three months after surgery to assess the patency of the ventriculostomy.

## Results

During the study period, 20 cases of LIAS were managed at our institution. The mean age at diagnosis was 40.8 years (range = 19-63 years.). The cohort comprised 12 males and eight females. The median follow-up period was three years (range = 1 month to 21 years).

One patient was lost to follow-up. The very variable follow-up (one month in three cases) resulted from a patient’s care being transferred to another neurosurgical unit one month after surgery, while another case was discharged one month postoperatively following complete resolution of their symptoms. The third case was referred back to the referring neurology services following normal ICP measurements. Table 1 summarises our cohort of patients.

**Table 1:** Demographics, management, and outcomes of our patient cohort. *: Evans index calculated from the earliest available diagnostic cranial imaging. ETV = endoscopic third ventriculostomy; RTA = road traffic accident; VPS = ventriculoperitoneal shunt; ICP = intracranial pressure; PMH = past medical history

Case number	Age at diagnosis/Gender	Year of diagnosis	Symptoms/Length of complaint	Neurological finding	Investigation	Evans index^*^	Treatment modality	Outcome	Length of follow-up from the intervention
1	19 years old/Female	1996	Headache (main, 2 weeks, recent mild head trauma)	None	CT, recent MRI	No available diagnostic imaging	VPS in 1996	Initial improvement, recurrent symptoms: VPS revision ×2, ETV in 2014	20 years
2	21 years old/Female	1996	Chronic headache (main), blurred vision	Optic atrophy	CT	0.37	VPS in 1996	Initially improved, shunt revisions ×3	24 years
3	57 years old/Male	1997	Memory changes (main), headache (chronic since 1997), history of head trauma at age 18	None	CT	0.37	Initial conservative, VPS in 2006	Initial improvement, 6 months mild headache, >6 months shunt adjusted	9 years
4	33 years old/Male	1999	Chronic headache (main), visual memory	Papilloedema	CT	No available diagnostic imaging	VPS in 1999	Initial improvement, multiple revisions and slit ventricle syndrome	21 years
5	63 years old/Female	2000	Seizures, memory, PMH of epilepsy	None	MRI	No imaging available	ETV in 2001, ETV success score: 90	Minimal improvement	2 years
6	58 years old/Male	2003	Chronic headache (main, 4 years), gait (main), urinary incontinence, visual, tinnitus	Optic atrophy	CT	0.44	VPS in 2003	Improved residual imbalance, hearing issues	17 years
7	56 years old/Male	2006	Chronic gait (history of tuberculosis meningitis as a kid, no residual abnormality)	None	CT	0.53	ICP monitoring: normal, conservative management	Symptoms unchanged	1 month
8	19 years old/Male	2008	Headache, acute (main)	Papilloedema	MRI	0.45	ETV in 2008, ETV success score: 90	Improved	1 month
9	55 years old/Female	2008	Chronic headache	None	CT	0.34	ETV in 2008, success score: 90	Improved	2 years
10	26 years old/Male	2011	Chronic headache (1 year), blurred vision	Papilloedema	MRI	0.36	ETV in 2011, success score: 90	Improved, later headache (2 years, ICP normal)	3 years
11	20 years old/Female	2012	Acute headache (main), blurred vision	Papilloedema	MRI	0.37	ETV in 2012, success score: 90	Improved initially, lost for long follow-up	5 months, then care transferred to another trust
12	45 years old/Male	2012	Chronic headache (main), blurred vision, memory disturbances, PMH of RTA	Diplopia, dyslexia	MRI	0.42	ETV in 2012, success score: 90	Partial improvement with marginal worsening of memory	1 year
13	31 years old/Female	2013	Acute headache (main), blurred vision, PMH of migraine, anxiety	None	MRI	0.28	ICP monitoring: normal, conservative	Persistent mild headache, lost for follow-up	1 year
14	44 years old/Male	2013	Chronic headache (9 months, main), blurred vision	None	MRI	0.36	ICP monitoring: normal, conservative	Symptoms improved	1 month
15	31 years old/Female	2014	Chronic headache (main), blurred vision	Papilloedema	MRI	0.4	ETV in 2014, success score: 90	Improved	6 months
16	48 years old/Male	2014	Chronic headache (main), blurred vision, PMH of HTN	None	MRI	0.37	Conservative, ICP 2015	Persistent symptoms, listed for VPS placement	6 years
17	43 years old/Male	2015	Acute headache (main), blurred vision, diplopia, memory loss, PMH of depression	None	MRI	0.36	ETV in 2015, success score: 90	Improved, late headache (>6 months, normal ICP)	3 years
18	56 years old/Male	2016	Chronic headache (main), recent RTA 2016	None	MRI	0.37	Conservative	Symptoms settled	1 year
19	49 years old/Female	2016	Chronic headache (main), urinary incontinence, PMH of RTA in 2016	None	MRI	0.42	Initial conservative, VPS in 2019	Improved	4 years
20	42 years old/Male	2018	Acute headache (main)	None	MRI	0.41	ETV in 2018, success score: 90	Failure, VPS inserted in 1 year	3 years

Clinical presentation

Headache was present in 17 cases and the main presenting feature in 16; 12 patients reported a history of chronic headaches. Three patients had a concurrent diagnosis of migraine. Other presenting symptoms included visual deterioration in 11 (55%) cases (chronic visual impairment was reported in seven cases), and five patients were found to have papilloedema on fundoscopy. Of those suffering from chronic visual impairment (>6 months; n = 7), two patients were found to have optic atrophy. Cognitive decline was present in five cases (for three cases it was the presenting symptom), gait disturbance in four cases (predominant feature in two cases), and urinary symptoms in two cases.

Past medical history was only of significance in two patients; one patient had a history of childhood tuberculous meningitis without evidence of hydrocephalus until presentation in adulthood (aged 56 years old) and another had a history of mild head trauma (without evidence of intracranial haemorrhage), which preceded the hydrocephalic symptoms by several weeks.

Radiological diagnosis

**Figure 1:**
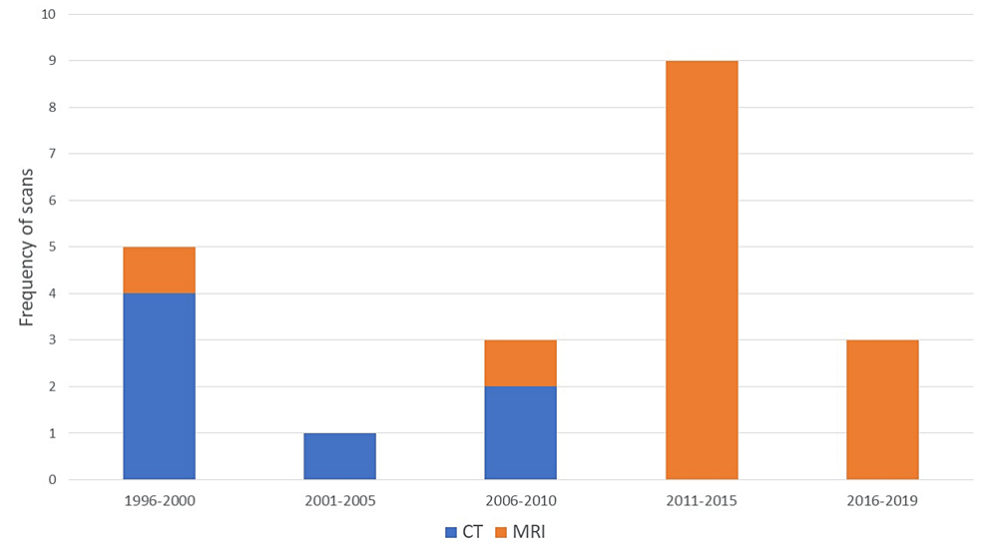
Imaging modalities employed according to the year of initial diagnosis.

Figure 1 summarises the frequency and type of imaging modalities used according to the year of diagnosis.

**Figure 2:**
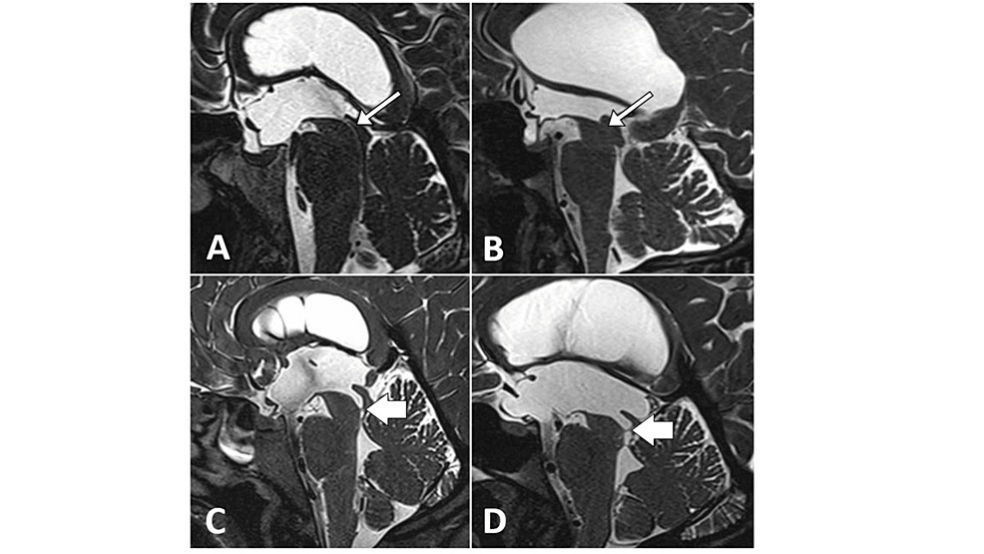
Preoperative CISS sequence MRI sagittal sections in four patients. (A, B) MRI for patient numbers 12 and 15 and (C, D) 17 and 20. The thin arrow points to aqueductal stenosis, and the thick arrow points to the aqueductal web. CISS = constructive interference in steady state

The diagnosis was confirmed radiologically by MRI in 13 (65%) cases and CT in seven (35%) cases. For cases with chronic headaches, there were no previous images to compare ventricular size. Of the cases diagnosed before 2008 (n = 7), the majority (n = 6, 86%) received a diagnosis based on the results of a CT scan. The Evans ratio of the available diagnostic scans ranged between 0.28 and 0.53 (mean = 0.39) (Figure 2).

Management

**Figure 3:**
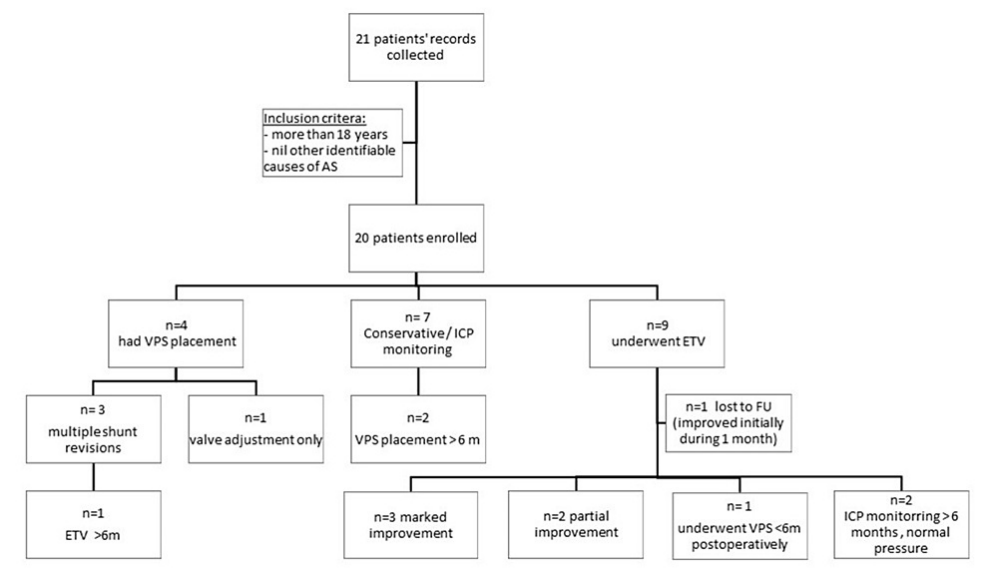
Flowchart of treatment modalities, revision, and conversion. AS = aqueductal stenosis; ETV = endoscopic third ventriculostomy; VPS = ventriculoperitoneal shunt; ICP = intracranial pressure; FU = follow-up

Nine patients underwent an ETV as the primary procedure (eight were treated after 2008), four patients received a VPS as initial management (all before 2008), and conservative management was employed in seven cases. In four of these, ICP was deemed normal following a period (24-48 hours) of ICP monitoring (Figure 3).

Treatment decisions appeared to vary based on the era of the patient’s presentation. For example, patients who presented before 2008 were more likely to undergo CT head imaging and subsequent VPS treatment, whereas, from 2008 onwards, MRI was the preferred imaging modality and ETV ± ICP monitoring became the first-line management option for LIAS patients (Figure 1).

Outcomes

Two patients initially treated conservatively subsequently underwent VPS placement due to unresolved symptoms. One patient who underwent an ETV required further treatment in the form of VPS placement during the first six months of follow-up, and three underwent ICP monitoring after six months for recurrent symptoms which revealed normal pressures.

In the ETV group, six patients underwent follow-up head MRIs showing patent ventriculostomy with fewer dilated ventricles. One had a CT head scan in the follow-up period showing smaller ventricles. One patient had no postoperative imaging. Two underwent postoperative MRI which showed non-functioning ventriculostomies. One underwent a VPS placement subsequently. The other patient’s care was transferred to another trust.

Of the six patients who received a VPS either as the initial or subsequent treatment, four necessitated revisions and/or required valve adjustment within 12 months of surgery. Three required at least two revisions, and a fourth patient necessitated a valve adjustment. One patient underwent an ETV a few months after her last VPS revision (Figure 3).

## Discussion

LIAS is a type of obstructive hydrocephalus in which CSF flow is limited or blocked at the level of the cerebral aqueduct with no other pathological cause identified (tumours, infection, or bleeding) typically presenting in adulthood. The obstruction (either caused by aqueductal narrowing or due to a web at the aqueduct level) is likely to be congenital. The patient chronically compensates until their presentation later in life. In our case series, all patients presented at >18 years old with a mean age of 40.8 years.

In cases of LIAS, the underlying cause of AS is still unexplained, as is the reason for a late presentation. It has been postulated that the pathophysiology might be malformative or inflammatory, the latter arising due to an abnormal proliferation of aqueductal subependymal glia [11]. The characteristic adult onset may occur due to decompensation of CSF flow. In our series, 12 patients had a history of chronic headaches; however, all patients had their scans beyond the age of 18 years, with no previous history of intracranial pathology or hydrocephalus. Only two of our patients had relevant history. One had tuberculosis meningitis as a child, without residual intracranial complications, and the other suffered head trauma.

Literature dating back to the early 1970s attempts to describe the phenomenon of triventricular hydrocephalus in adults. Aqueductal flow dysfunction was suspected in hydrocephalic patients with enlargement of the third and lateral ventricles on skull ventriculography or pneumoencephalography. Harrison et al. (1974) and Little et al. (1975) reported 72 cases of suspected LIAS in which the mean age was 28 years. Little et al. (1975) reported some success in the treatment of symptoms following shunting [2,12].

From 2001 onwards, coinciding with the advent of MRI and the advancement of endoscopic treatment modalities, more case series of LIAS have been reported, the largest of which was published by Fukuhara and Luciano in 2001. They reported on 31 patients (mean age = 46.7 years), all of whom underwent ETV with a reported significant improvement in 26 cases [9]. The earlier series had different descriptions for the phenomenon, including non-tumoural and benign AS in adults [1,6].

We have undertaken a literature review including published cases that are searchable via PubMed using the following keywords: adult-onset, late-onset, aqueductal stenosis, and obstructive hydrocephalus. The review included the age, gender, clinical presentation, treatment modality, and outcome. Some literature describes endoscopic aqueductoplasty as an efficacious treatment modality (Table 2) [13-16].

**Table 2:** A literature review of cases with LIAS and LAMO demonstrating demographics and management listed in chronological order. LIAS = late-onset idiopathic aqueductal stenosis; LAMO = late-onset aqueductal membranous occlusion; NA = not available; FIESTA = fast imaging employing steady-state acquisition; EA = endoscopic aqueducatoplasty; NPH = normal-pressure hydrocephalus; LOC = loss of consciousness; FU = follow-up

Study authors	Study year	Sample size	Mean age in years	Main presenting symptoms	Average duration	Neurological findings	Investigations	Treatment modality	Outcome
Harrison et al. [12]	1974	55 (28 M, 27 F)	39 (range = 16–62)	Headache: 20, visual: 11, gait: 9, epilepsy: 5, others (mental deterioration, endocrine, incontinence, falls)	2 days to 40 years	Papilledema: 29, optic pallor: 7, optic atrophy: 12, intellectual changes: 12, ataxia: 16, motor/pyramidal: 24, nystagmus: 4	EEG: 32, Plain X-ray of the skull: 53, carotid angiography: 13, pneumoencephalography: 16, ventriculography: 28	NA	NA
Little et al. [2]	1975	17 (10 M, 7 F)	28 (range = 19–52)	Headache: 4, visual: 3, seizures: 4, CSF rhinorrhoea: 4, hormonal: 2, gait/motor: 3	6 years	Normal: 5, visual: 6 (papilledema/optic atrophy/field defects), gait/motor disturbances: 6	EEG, skull X-ray, CSF analysis, right retrograde brachial angiography, air ventriculography	Shunt: 17, CSF fistula repair: 2	Mean FU duration: 10 years, 7 recovered, occasional headache: 2, seizures persisted: 2, Seizures reduced: 2
Fukuhara and Luciano [9]	2001	31 (13 M, 18 F	46.7 (range = 12.9–86)	Headache alone: 12, visual: 4, NPH alone: 11, both NPH and headache: 2	Set up clinical criteria for LIAS; acute (<1 month): 1 subacute (1–6 months): 1 chronic (longer than 6 months): 29	Papilledema: 7	Cine phase-contrast MRI: 26, MRI only: 5	ETV: 31, repeat ETV: 1, VPS after ETV: 5	Mean FU duration: 26.2 months, significant improvement: 26, temporary improvement: 4, intraoperative bleeding: 1
Burtscher et al. [17]	2002	6 (3 M, 3 F)	30.5 (range = 25–60)	Headache: 5, gait : 3	5.7 (1–12 months)	Papilledema: 1, amenorrhea: 2	MRI preoperative: 1, 6, 12, and 24 weeks postoperative (including spin echo, cardiac-gated cross-sectional phase contrast, and 3D flash sequences), neuropsychological assessment	ETV: 6	Headache improved in 5, amenorrhea improved in 2, neuropsychological assessment performed in 2 returned to normal in 3, good recovery in 1 persistent deficit
Bateman [7]	2007	21 (11 M, 10 F)	52	Headache: 12, NPH: 9	NA	NA	MRI 1.5 T (examining ventricular enlargement, sulcal compression, total blood in-flow, superior sagittal/straight sinus outflow, arteriovenous delay (AVD), and the extent of collateral venous flow)	NA	NA
Matsuda et al. [14]	2011	1 M	57	Gait and memory disturbances	1 year	Bradykinesia and impairment of postural reflex	MRI, FIESTA	ETV + EA	Improved
Chen et al [13]	2013	6 (2 M, 4 F)	Range = 20–67	Headache/vomiting: 5, blurred vision and LOC: 1	NA	NA	MRI	EA	Improved
Locatelli et al. [18]	2014	13 (9 M, 4 F)	56.8 ( range = 22–72)	Headache: 2; gait:8, cognitive decline: 2, visual: 1	Fukuhara I: 1, Fukuhara II: 4, Fukuhara III: 8	Papilloedema: 4, gait/motor: 10	MRI: 13, MRI Cine phase contrast: 10	ETV: 13	Mean FU: 30.6 (3–52 months), improved symptoms in 13, persistent urinary symptoms in 3, stable cognition in 2
Kita et al. [15]	2014	1 M	17	Headache and mild cognitive impairment	Since adolescence	Mild intellectual disability	MRI, IQ test		Improved headache and intellectual functions
Bradley et al. [8]	2015	15	NA	NA	NA	NA	MRI (FIESTA, ADC)	NA	NA
Terada et al. [16]	2020	1 (LAMO)	NA	Headache, loss of consciousness	Acute	Mild papilledema	MRI (spin echo, 3D DRIVE, phase contrast vine)	ETV + EA	Improved

Notably, Fukahara and Luciano employed MRI primarily in the diagnosis of LIAS, as well as the usage of ETV as the first-line treatment. That trend was also observed in subsequent studies.

In our series, a significant proportion of the cases presented (60%) with chronic symptoms greater than six months before diagnosis. Symptoms included headache, visual disturbance, memory deterioration, cognitive impairment, and urinary symptoms. In six (30%) patients, a history of chronic headaches was reported, three of whom had received a prior diagnosis of migraine. Our findings were in keeping with the series reported by Fukuhara and Luciano who classified the clinical presentation of LIAS into acute (<1 month), subacute (1-6 months), and chronic (>6 months), with younger patients typically presenting with headache [9].

In our cohort, post-2008, we observed a change in practice related to diagnostic imaging and management. Specifically, patients were more consistently diagnosed following MRI, and ETV became the procedure of choice. ETV successfully treated eight patients (89% of the ETV group) over an average follow-up period of 1.5 years. In the single case of ETV failure, shunt insertion was required. Several studies have confirmed the effectiveness of ETV for patients with LIAS as most obtain a complete recovery of the symptoms; however, ventriculomegaly usually persists on follow-up imaging [17,18]. Our findings corroborate the high success rate of ETV for LIAS observed by others. These findings also validate high ETV success scores calculated retrospectively for our cohort, with all patients scoring 90/100. All patients receiving a VPS as the primary treatment required further intervention in the form of either shunt revisions, ICP monitoring, or shunt valve adjustment within 12 months of surgery.

Of the cohort, 12/20 patients required at least one additional intervention, including ICP monitoring, valve adjustment or VPS revision, during the follow-up period. A single patient suffering from chronic symptoms reported worsening of their condition, specifically gait impairment and hearing loss. This suggests that as with other forms of hydrocephalus, close follow-up is required.

Conclusions drawn from our observation study are limited by its sample size which prevents a meaningful statistical comparative analysis. Moreover, the study conclusions are based on retrospective analyses and are limited by cohort selection bias. Seven patients in our cohort received a diagnosis of LIAS based on CT which is inferior to MRI, and it may be that subtle underlying pathology was not observed; however, progressive pathology was not identified in these cases during follow-up. Although not performed in all cases, formal neuropsychological assessment at the time of diagnosis and during follow-up which, if correlated with follow-up imaging, would have provided additional valuable information. Some elderly patients with LIAS may have received a diagnosis of normal pressure hydrocephalus, so our cohort may be an underestimate of the true number of patients with the condition. Moreover, the very variable follow-up periods, which included some short follow-up periods, skewed the data analysis and interpretation of the treatment outcomes.

We advocate further prospective analysis of this uncommon condition via multi-centre collaborative data collection efforts. Unanswered questions remain such as what mechanisms lead to the acute and chronic presentations observed in our and other cohorts, and why does it present in adulthood? Furthermore, what are the long-term outcomes for patients receiving either form of CSF diversion surgery? Moreover, a detailed neuropsychological assessment of such patients before and following intervention would be helpful to determine the potential psychological effects of treatment and evaluate its utility as a follow-up tool.

## Conclusions

LIAS is an uncommon form of adult-onset obstructive hydrocephalus, which may present with acute or chronic symptoms and characteristic MRI findings. Clinicians should be mindful of the condition, particularly in those suffering from headaches, which, if missed, may lead to devastating neurological sequelae. The condition is amenable to treatment with an ETV, as demonstrated in our cohort, due to its obstructive nature and associated ventriculomegaly, which permits endoscopic ventricular access. CSF shunting, while treating the hydrocephalus, was associated with a high rate of further interventions in this cohort. Collaborative multi-centre efforts to amass further data may facilitate a better understanding of LIAS and provide answers to some of the remaining questions.

## References

[1] Nag TK, Falconer MA (1966). Non-tumoral stenosis of the aqueduct in adults. Br Med J.

[2] Little JR, Houser OW, MacCarty CS (1975). Clinical manifestations of aqueductal stenosis in adults. J Neurosurg.

[3] Cinalli G, Spennato P, Nastro A (2011). Hydrocephalus in aqueductal stenosis. Childs Nerv Syst.

[4] Spennato P, Tazi S, Bekaert O, Cinalli G, Decq P (2013). Endoscopic third ventriculostomy for idiopathic aqueductal stenosis. World Neurosurg.

[5] Jellinger G (1986). Anatomopathology of non-tumoral aqueductal stenosis. J Neurosurg Sci.

[6] Vanneste J, Hyman R (1986). Non-tumoural aqueduct stenosis and normal pressure hydrocephalus in the elderly. J Neurol Neurosurg Psychiatry.

[7] Bateman GA (2007). Magnetic resonance imaging quantification of compliance and collateral flow in late-onset idiopathic aqueductal stenosis: venous pathophysiology revisited. J Neurosurg.

[8] Bradley WG, Abdihalim M, Almutairi A (2015). Adult onset aqueductal stenosis may become symptomatic due to deep white matter ischemia. Fluids Barriers CNS.

[9] Fukuhara T, Luciano MG (2001). Clinical features of late-onset idiopathic aqueductal stenosis. Surg Neurol.

[10] Kehler U, Regelsberger J, Gliemroth J, Westphal M (2006). Outcome prediction of third ventriculostomy: a proposed hydrocephalus grading system. Minim Invasive Neurosurg.

[11] Dandy WE (1945). Diagnosis and treatment of strictures of the aqueduct of Sylvius (causing hydrocephalus). Arch Surg.

[12] Harrison MJ, Robert CM, Uttley D (1974). Benign aqueduct stenosis in adults. J Neurol Neurosurg Psychiatry.

[13] Chen G, Zheng J, Xiao Q, Liu Y (2013). Application of phase-contrast cine magnetic resonance imaging in endoscopic aqueductoplasty. Exp Ther Med.

[14] Matsuda M, Shibuya S, Oikawa T, Murakami K, Mochizuki H (2011). [A case of late-onset aqueductal membranous occlusion and a successful treatment with neuro-endoscopic surgery]. Rinsho Shinkeigaku.

[15] Kita D, Hayashi Y, Kitabayashi T, Kinoshita M, Okajima M, Taniguchi T, Hamada J (2014). Detection of the development of late-onset idiopathic aqueductal stenosis (LIAS) by chronological magnetic resonance imaging: a case report. Childs Nerv Syst.

[16] Terada Y, Yamamoto M, Motoie R, Matsui Y, Katsuki T, Mori N, Hashimoto K (2020). Hydrocephalus resulting from late-onset aqueductal membranous occlusion: a case report and review of the literature. World Neurosurg.

[17] Burtscher J, Bartha L, Twerdy K, Eisner W, Benke T (2003). Effect of endoscopic third ventriculostomy on neuropsychological outcome in late onset idiopathic aqueduct stenosis: a prospective study. J Neurol Neurosurg Psychiatry.

[18] Locatelli M, Draghi R, DI Cristofori A (2014). Third ventriculostomy in late-onset idiopathic aqueductal stenosis treatment: a focus on clinical presentation and radiological diagnosis. Neurol Med Chir (Tokyo).

